# Surface Analysis of Lithium Disilicate Ceramics After Use of Charcoal-Containing Toothpastes

**DOI:** 10.3390/jfb16050183

**Published:** 2025-05-15

**Authors:** Franciele Floriani, Bayaan Jabr, Silvia Rojas-Rueda, Rene Garcia-Contreras, Carlos A. Jurado, Abdulrahman Alshabib

**Affiliations:** 1 Department of Prosthodontics, College of Dentistry, The University of Iowa, Iowa City, IA 52242, USA; 2 College of Dentistry, The University of Iowa, Iowa City, IA 52242, USA; 3 Division of Dental Biomaterials, Department of Clinical and Community Sciences, School of Dentistry, The University of Alabama at Birmingham, Birmingham, AL 35233, USA; 4 Interdisciplinary Research Laboratory, Nanostructures, and Biomaterials Area, National School of Higher Studies (ENES), National Autonomous University of Mexico (UNAM), Leon 37684, Mexico; 5 Division of Operative Dentistry, Department of General Dentistry, College of Dentistry, The University of Tennessee Health Science Center, Memphis, TN 38103, USA; 6 School of Dental Medicine, Ponce Health Sciences University, Ponce, PR 00732, USA; 7 Department of Restorative Dentistry, College of Dentistry, King Saud University, Riyadh 11545, Saudi Arabia

**Keywords:** charcoal, toothpaste, lithium disilicate, ceramics, roughness

## Abstract

Background: This study evaluated the effect of charcoal-containing toothpaste on the surface roughness of CAD/CAM lithium disilicate ceramic (e.max CAD) after simulated toothbrushing. Methods: Forty-eight e.max CAD ceramic specimens were divided into four groups (n = 12) and subjected to 18,000 brushing cycles using a toothbrushing simulator. The groups included Crest 3D White Charcoal, Colgate Optic White with Charcoal, Arm & Hammer Charcoal White, and a control group (conventional toothpaste). Surface roughness was measured with a profilometer before and after brushing, and scanning electron microscopy (SEM) was used for topographical analysis. Statistical analysis was performed using the Kruskal–Wallis test and post hoc comparisons. Results: Significant differences in surface roughness were found among the groups (*p* < 0.001). The mean roughness values were 540.70 ± 21.68 µm (Control), 294.88 ± 11.49 µm (Crest 3D White Charcoal), 1157.00 ± 52.85 µm (Colgate Optic White with Charcoal), and 593.37 ± 37.69 µm (Arm & Hammer Charcoal White). Post hoc analysis showed that Colgate Optic White with Charcoal had the highest roughness, which was significantly different from all other groups (*p* < 0.001). SEM analysis revealed severe surface degradation with Colgate Optic White with Charcoal, while Crest 3D White Charcoal caused minimal changes. Conclusions: Charcoal-containing toothpastes vary in abrasiveness, with Colgate Optic White with Charcoal causing the most significant surface roughness and damage to lithium disilicate ceramics.

## 1. Introduction

The literature underscores the pivotal role of toothpaste in preventing dental caries and gingival diseases [1,2]. Toothbrushing with toothpaste has become a universal standard for maintaining oral hygiene, making toothpaste an essential component of daily oral care routines.

Traditional toothpastes are formulated with fluoride compounds, including sodium fluoride, amine fluoride, stannous fluoride, and sodium monofluorophosphate. These fluoride agents have been widely recognized for their efficacy in preventing dental caries and strengthening enamel [2,3]. However, in response to consumer preferences, fluoride-free alternatives have emerged. These toothpastes often incorporate components such as calcium phosphates and xylitol, which also offer protective benefits against caries by promoting enamel remineralization and reducing bacterial activity [4,5]. In recent years, there has been a significant rise in patient demand for products that not only promote oral health but also enhance aesthetic outcomes, such as whiter and healthier-looking teeth. To meet these evolving expectations, manufacturers have introduced a variety of toothpastes with specialized compositions [6,7]. Patients today have access to an extensive range of toothpaste options tailored to diverse needs and preferences. These include traditional fluoride-based formulations, whitening toothpastes designed to remove surface stains [8,9], fluoride-free variants, herbal-based toothpastes featuring natural extracts [10], sea salt-enriched toothpastes that claim to enhance cleaning effectiveness [11], and charcoal-infused toothpastes marketed for their whitening and detoxifying properties [12]. Given the plethora of choices, it is imperative for patients to carefully evaluate the type of toothpaste they use. Considerations should include individual oral health needs, sensitivity to ingredients, and alignment with personal aesthetic goals. Dental professionals play a critical role in guiding patients toward selecting the most appropriate toothpaste for their specific requirements.

The increasing demand for aesthetic dental restorations has led to the widespread use of CAD/CAM lithium disilicate ceramics (e.max CAD), prized for their outstanding mechanical strength, natural translucency, and close resemblance to natural teeth. These attributes make lithium disilicate ceramics a popular choice for various restorations, such as crowns, veneers, and bridges. Preserving the surface integrity of these restorations is critical for maintaining their long-term functionality and aesthetic appeal. Surface roughness, in particular, significantly influences key properties like wear resistance, staining susceptibility, and bacterial adhesion. Higher surface roughness can lead to increased discoloration and bacterial accumulation, potentially resulting in premature failure and the need for replacement [13]. The ceramic surface can degrade over time due to factors like dietary habits, acidic beverages, oral hygiene routines, and the abrasiveness of toothpaste. Each of these factors contributes to the gradual deterioration of the ceramic surface [14]. Among these, toothpaste formulations play a pivotal role in determining surface roughness. Charcoal-containing toothpastes, which are popular for their claimed whitening and stain removal properties, have raised concerns regarding their abrasivity [15]. Their abrasive nature may accelerate the wear of the ceramic surface, increasing roughness and potentially diminishing the restoration’s aesthetic and functional performance. As charcoal-based toothpastes gain popularity, it is crucial to investigate their effects on dental materials, particularly CAD/CAM ceramics, to ensure they do not inadvertently compromise the longevity of these restorations.

Charcoal-based toothpastes have become increasingly popular in recent years, largely driven by their marketed benefits for whitening and stain removal [16]. Many manufacturers promote activated charcoal as an effective agent for eliminating extrinsic stains from the teeth, with the added claim that it does so without causing damage to enamel or dental restorations. Despite these claims, there is still a lack of definitive scientific evidence regarding the impact of charcoal toothpaste on dental materials, especially CAD/CAM ceramics [17]. While some studies have reported that charcoal toothpaste may have a higher abrasivity compared to traditional fluoride-containing toothpastes, concerns have arisen about its potential to cause increased surface roughness and accelerated wear on ceramic restorations [18,19]. This heightened abrasivity may be due to the nature of activated charcoal particles, which can be coarser than the mild abrasives typically found in conventional toothpaste formulations. The long-term consequences of regular use of charcoal-based toothpastes on the integrity and longevity of CAD/CAM ceramics remain uncertain, necessitating further investigation to fully understand the implications of their use on restorative dental materials. As such, it is crucial to critically evaluate the balance between the aesthetic benefits promoted by these toothpastes and their potential adverse effects on dental restorations.

The composition of dental ceramics is a key factor influencing their mechanical and optical properties, which directly affect their clinical performance and durability [20]. Dental ceramics are generally categorized into glass-based, glass-infiltrated, and polycrystalline ceramics, each with unique attributes [21]. Glass-based ceramics, including feldspathic porcelain and lithium disilicate, are renowned for their excellent aesthetics due to their translucency but typically have lower mechanical strength compared to other types [22]. Glass-infiltrated ceramics integrate crystalline phases within a glass matrix, offering enhanced strength while retaining some translucency [23]. In contrast, polycrystalline ceramics, such as zirconia, provide exceptional strength and fracture resistance but lack the high translucency of glass-based materials [23]. Among these, lithium disilicate emerges as a versatile choice due to its well-balanced properties, including high flexural strength (~400 MPa), fracture toughness, and desirable optical characteristics [23].

Lithium disilicate (Li_2_Si_2_O_5_) is a silica-based glass-ceramic that undergoes a controlled crystallization process, forming an interlocking network of needle-like lithium disilicate crystals within a glass matrix [24]. This microstructure enhances its mechanical strength and resistance to crack propagation, making it a preferred material for CAD/CAM restorations, such as veneers, crowns, and implant-supported prostheses [25]. Furthermore, its high translucency and superior shade-matching capabilities enable outstanding aesthetic results that closely mimic natural teeth [26]. However, like other glass-based ceramics, lithium disilicate’s surface properties are susceptible to mechanical and chemical interactions within the oral environment, including abrasion from toothpastes and exposure to dietary acids [27,28]. The potential impact of prolonged brushing with charcoal toothpaste on lithium disilicate ceramics remains underexplored, underscoring the necessity for further research in this area.

Surface roughness plays a critical role in the clinical performance of dental ceramics, particularly in regard to their durability, aesthetic appeal, and functionality [29]. An increase in surface roughness can have several negative effects, most notably reducing the gloss and overall appearance of the restoration, making it more susceptible to staining over time [30]. Staining can detract from the aesthetic qualities of the restoration, leading to patient dissatisfaction. Additionally, roughened surfaces provide an ideal environment for bacterial adhesion, which can significantly increase the risk of plaque accumulation and the development of secondary caries. This accumulation of plaque not only compromises oral hygiene but also contributes to the degradation of the restoration and surrounding tooth structure. Given these concerns, it is crucial to ensure that toothpaste formulations do not negatively impact the integrity of CAD/CAM lithium disilicate surfaces. This is especially important for maintaining the longevity and functionality of dental restorations, as surface integrity is directly linked to their performance and aesthetic outcomes. While prior research has explored various polishing techniques and their effectiveness in preserving smooth ceramic surfaces, there remains a significant gap in the literature regarding the direct effects of charcoal-containing toothpaste on CAD/CAM ceramics. Given the increasing popularity of charcoal-based toothpastes, further investigation is needed to understand their potential impact on the surface properties and overall longevity of these restorations.

The present study evaluated the effect of charcoal-containing toothpaste on the surface roughness of CAD/CAM lithium disilicate ceramic (e.max CAD) after simulated toothbrushing. A total of 48 ceramic specimens underwent 18,000 brushing cycles, simulating approximately two years of clinical use. The specimens were divided into four groups (n = 12 per group) based on the toothpaste used: Crest 3D White Charcoal (Batch No: L9267A01), Colgate Optic White with Charcoal (Batch No: 1234CW01), Arm & Hammer Charcoal White (Batch No: AH785CX), and a Control group using a conventional fluoride toothpaste, Colgate Cavity Protection (Batch No: C1023FL), were used in this study. Standardized soft-bristled toothbrush heads were used to minimize variability in abrasiveness, ensuring uniform brushing conditions across all specimens.

## 2. Materials and Methods

### 2.1. Specimen Preparation

A total of 48 CAD/CAM lithium disilicate ceramic specimens (e.max CAD, Ivoclar Vivadent, Schaan, Liechtenstein) were used for this study. These specimens were carefully selected to ensure consistency and accuracy in the experimental outcomes. To achieve uniformity, each specimen was precisely standardized to dimensions of 12 mm × 10 mm × 1.5 mm. This step was performed using a high-precision cutting machine (Isomet, Buehler, Lake Bluff, IL, USA), ensuring that each specimen had identical size and shape. After sectioning, the specimens underwent a crystallization process, which was conducted following the manufacturer’s recommended protocol. This crucial step allowed the specimens to develop the desired mechanical properties and microstructure that are characteristic of e.max CAD lithium disilicate ceramics. The crystallization process is essential for enhancing the material’s strength, durability, and overall performance, ensuring that the specimens closely resemble the characteristics of clinically used restorations.

Following the crystallization, all specimens were subjected to a standardized polishing procedure. This step was necessary to achieve a smooth baseline surface, ensuring that accurate and reproducible roughness measurements could be taken. The polishing procedure was carefully controlled to avoid introducing any additional surface alterations that could affect the study’s results. By establishing a smooth and consistent starting point, the study was able to reliably measure the changes in surface roughness resulting from exposure to the different toothpaste formulations.

### 2.2. Grouping and Brushing Simulation

The specimens were randomly assigned into four groups, with each group consisting of 12 specimens (n = 12), based on the toothpaste formulation used. The four groups included the following: Crest 3D White Charcoal, Colgate Optic White with Charcoal, Arm & Hammer Charcoal White, and the Control group, which used a conventional toothpaste without charcoal. Random assignment of the specimens to these groups helped eliminate any bias and ensured that the results were statistically valid and representative of the effects of each toothpaste formulation. Each specimen was subjected to a total of 18,000 brushing cycles, a process designed to simulate approximately two years of typical clinical brushing. To maintain consistent and controlled brushing conditions, distilled water was used as the brushing medium to mimic the presence of saliva in the oral environment. The toothbrushing cycles were performed using a high-precision toothbrushing simulator (V8 Cross Brushing Machine, Germany) specifically designed to replicate standardized brushing movements under controlled conditions. This machine ensures uniform brushing conditions, providing a reliable and reproducible environment for evaluating the effects of different toothpaste formulations on the surface roughness of the specimens. By simulating two years of brushing, the study aimed to assess the long-term impact of charcoal-based toothpastes on dental ceramics, ensuring that the results reflected real-world use. This method also allowed for accurate comparisons between the different toothpaste groups under standardized conditions.

### 2.3. Brushing Simulation Parameters

Each specimen was subjected to 18,000 brushing cycles—equivalent to approximately two years of twice-daily toothbrushing—using a V8 Cross Brushing Machine (Germany) to ensure uniform, standardized conditions across all groups. Standardized soft-bristled toothbrush heads were employed to minimize variability in abrasiveness. A toothpaste slurry was prepared in a 1:2 ratio of toothpaste to distilled water to replicate the dilution that naturally occurs during manual brushing. The simulator applied a controlled force of 2 N to each specimen and was programmed to perform 120 strokes per minute, thereby mimicking typical brushing pressure and speed. After completion of the brushing cycles, specimens were thoroughly rinsed with distilled water to remove residual slurry and debris.

**Figure 1:**
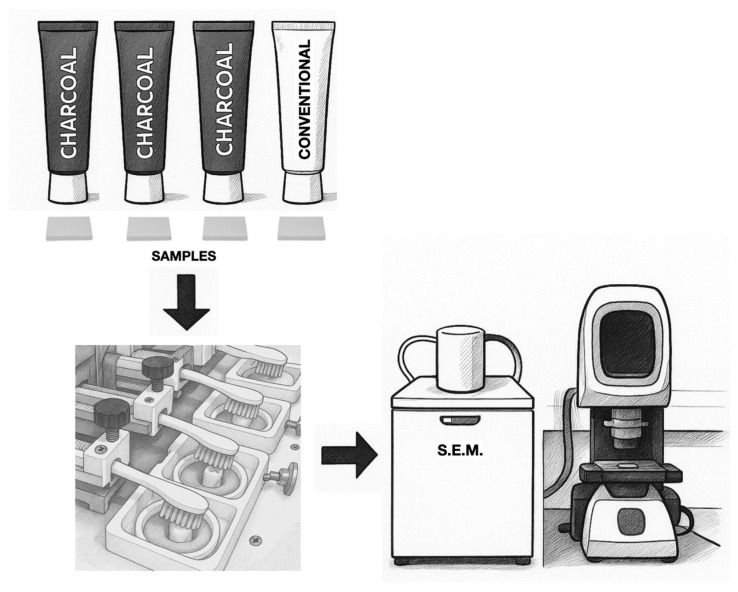
Experimental set-up for the samples receiving toothpaste followed by toothbrushing cycles, and finally, images of the profilometer and microcopy equipment.

Surface roughness was quantified post-brushing using a profilometer, with one measurement taken per specimen to calculate a reliable mean roughness value (Ra, in µm) for each group. In addition, scanning electron microscopy (SEM) at 250× magnification was performed on all specimens to visualize surface alterations induced by the charcoal-containing toothpastes. A detailed, step-by-step representation of the brushing protocol and subsequent analyses is provided in Figure 1.

### 2.4. Statistical Analysis

The data were presented as mean and standard deviation. Normality was assessed using the Shapiro–Wilk test, which revealed that the data were not normally distributed (*p* < 0.05). Therefore, the non-parametric Kruskal–Wallis test was employed to compare roughness measurements across the groups. Post hoc pairwise comparisons were performed using the Bonferroni correction to adjust for multiple tests. All statistical analyses were conducted using IBM SPSS Statistics Version 30, and graphs were generated using Microsoft Excel.

## 3. Results

The surface roughness measurements varied significantly across the groups (*p* < 0.001, Kruskal–Wallis test). The mean roughness values (±standard deviation) for each group were as follows: Control (540.70 ± 21.68 nm), Crest 3D White Charcoal (294.88 ± 11.49 nm), Colgate Optic White with Charcoal (1157.00 ± 52.85 nm), and Arm & Hammer Charcoal White (593.37 ± 37.69 nm) (Table 1).

Pairwise comparisons (Table 2) revealed that Crest 3D White Charcoal exhibited significantly lower surface roughness compared to both Arm & Hammer Charcoal White (*p* = 0.002) and Colgate Optic White with Charcoal (*p* < 0.001). This indicates that, among the charcoal-containing toothpastes, Crest 3D White Charcoal was the least abrasive, resulting in minimal surface damage to the lithium disilicate ceramic specimens. Interestingly, the roughness of the Crest 3D White Charcoal group did not differ significantly from the Control group, which used a conventional toothpaste without charcoal (*p* = 0.222), suggesting that the abrasive effect of Crest 3D White Charcoal is comparable to that of a traditional toothpaste on ceramic surfaces.

In contrast, Colgate Optic White with Charcoal demonstrated the highest surface roughness values, indicating that this formulation was the most abrasive among the tested products. The roughness observed with Colgate Optic White with Charcoal was significantly greater than that of the Control group (*p* = 0.002) and Arm & Hammer Charcoal White (*p* = 0.222). These findings suggest that the Colgate Optic White with Charcoal may have a higher abrasive potential, which could lead to more significant wear and surface degradation of dental ceramics over time. Despite the increased roughness, no significant difference in surface roughness was found between the Control group and Arm & Hammer Charcoal White (*p* = 0.701), indicating that the abrasivity of Arm & Hammer Charcoal White is similar to that of conventional toothpaste in terms of its effect on the ceramic surface.

To strengthen the statistical reporting and provide insight into the magnitude of observed differences, an effect size (η^2^) was calculated for the Kruskal–Wallis test. The result (η^2^ ≈ 0.80) indicates a large effect size, supporting the significance and relevance of the group differences in surface roughness.

The Colgate Optic White with Charcoal exhibited the highest surface roughness among all tested groups, followed by Arm & Hammer Charcoal White, Control, and Crest 3D White Charcoal, with the latter showing the lowest roughness values. These results indicate that the type of toothpaste plays a significant role in affecting the surface roughness of CAD/CAM lithium disilicate ceramics, with Colgate Optic White with Charcoal demonstrating the greatest abrasive effect.

**Figure 2:**
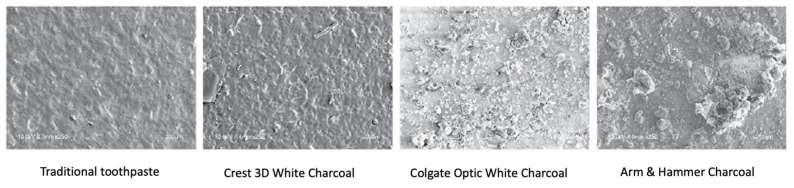
SEM images of CAD/CAM lithium disilicate ceramic surfaces after toothbrushing with different toothpastes (magnification ×250).

The scanning electron microscopy (SEM) images of the samples are shown in Figure 2 at 250× magnification. These images provide a detailed view of the surface changes on the lithium disilicate ceramics after exposure to various charcoal-containing toothpastes. The SEM analysis revealed significant surface roughness for the Colgate Optic White with Charcoal toothpaste, which exhibited noticeable abrasions and grooves on the ceramic surface. This was the most pronounced effect observed among the tested toothpastes, indicating that the formulation of this particular toothpaste may have a higher abrasive potential compared to others. Following this, Arm & Hammer Charcoal White showed moderate surface roughness, although it was less severe than that observed with Colgate Optic White with Charcoal. Crest 3D White Charcoal displayed the smoothest surface among the charcoal toothpastes, with minimal surface alterations. This suggests that the abrasive properties of Crest 3D White Charcoal are less aggressive, making it potentially safer for use on dental ceramics. Lastly, the Control group, which used traditional toothpaste without charcoal, also demonstrated a relatively smooth surface, with only slight changes in roughness. This indicates that conventional toothpaste formulations are likely gentler on the surface of lithium disilicate ceramics compared to some charcoal-based alternatives. These findings highlight the varying degrees of abrasivity among charcoal-containing toothpastes and their potential effects on the longevity and aesthetic appeal of dental restorations.

## 4. Discussion

The present study aimed to evaluate the effect of charcoal-containing toothpaste on the surface roughness of CAD/CAM lithium disilicate ceramic (e.max CAD) after simulated toothbrushing. The results revealed significant differences in surface roughness among the groups tested (*p* < 0.001), leading to the rejection of the null hypothesis, which posited that no significant differences would be observed between lithium disilicate specimens exposed to different charcoal toothpastes after 18,000 brushing cycles. This outcome highlights the influence of different charcoal-based toothpaste formulations on the wear and integrity of dental ceramics. Among the toothpastes tested, Colgate Optic White with Charcoal demonstrated the highest surface roughness, with a mean value of 1157.00 ± 52.85 nm, indicating that this formulation caused the most significant surface degradation. In contrast, Crest 3D White Charcoal exhibited the lowest roughness values (294.88 ± 11.49 nm), which were similar to those observed in the Control group (540.70 ± 21.68 nm). These findings suggest that not all charcoal-containing toothpastes have the same impact on the surface of lithium disilicate ceramics, with some being less abrasive and more protective of the ceramic surface than others. The observed variation in surface roughness can be attributed to differences in the abrasivity of the formulations, which may depend on factors such as the type and concentration of charcoal, as well as the presence of other abrasive agents in the toothpaste.

Surface roughness plays a vital role in the clinical performance of lithium disilicate ceramics, affecting their wear resistance, biofilm adherence, and optical qualities. This study aligns with the work of Mahrous et al. [13], who demonstrated that surface treatments and polishing protocols significantly influence the surface roughness of CAD/CAM lithium disilicate ceramics. While their research focused primarily on the effects of post-fabrication polishing, the present study highlights the abrasive impact of daily oral hygiene products on ceramic restorations. Similarly, Al-Angari et al. [14] investigated how coffee and whitening agents affect the surface roughness of lithium disilicate ceramics, finding that whitening treatments increased roughness. These results are consistent with the current study’s findings on the effects of charcoal-based toothpaste formulations.

Previous investigations assessing the abrasive effects of charcoal toothpastes on dental materials have primarily focused on resin composites. In a study conducted by Bragança et al. [15], it was observed that charcoal-containing toothpaste led to increased surface roughness in resin composites, supporting the hypothesis that charcoal particles contribute to material degradation. The present study expands upon these findings by evaluating charcoal toothpaste-induced roughness in lithium disilicate ceramics. Notably, Colgate Optic White with Charcoal exhibited the highest roughness values, whereas Crest 3D White Charcoal demonstrated no significant difference from the Control group (*p* = 0.222), suggesting variability in the abrasive potential of different formulations.

Further supporting the present findings, Dionysopoulos et al. [16] investigated the influence of whitening toothpastes with different active agents on dentin abrasion, concluding that abrasiveness is highly dependent on toothpaste composition rather than its marketed whitening efficacy. This aligns with the observations of the present study, as certain charcoal-containing formulations resulted in substantial surface alterations, whereas others exhibited minimal impact. Similarly, Koroglu et al. [17] reported that toothbrushing with whitening toothpastes increased surface roughness in interim prosthodontic materials, further emphasizing the necessity of careful toothpaste selection for patients with ceramic-based restorations.

The findings of the present study align with those of Matzinger et al. [20], who emphasized the influence of material selection and brushing protocols on the wear performance of chairside CAD/CAM restorations. Their research highlighted how specific brushing techniques and abrasive forces could affect the longevity and surface integrity of CAD/CAM restorative materials. The current study builds on this foundation by demonstrating that the composition of toothpaste is an equally critical factor in determining long-term changes in ceramic surface topography. While Matzinger et al. primarily focused on wear performance under varying brushing conditions, this study expands the discussion by underscoring the role of toothpaste abrasiveness and chemical properties. Toothpaste formulations containing highly abrasive agents, such as charcoal or other whitening additives, were shown to cause significant surface alterations in dental ceramics. These changes include increased roughness and potential microstructural damage, which could compromise both the aesthetics and functional durability of the restorations over time. Additionally, the present study highlights the interplay between brushing protocols and toothpaste composition, suggesting that even mild abrasives may exacerbate wear when combined with high brushing pressure or frequency. These findings reinforce the importance of careful material selection—not only for restorative materials but also for adjunctive oral hygiene products like toothpaste—to optimize the long-term performance of dental restorations. The insights from this study are particularly valuable for clinicians and patients aiming to balance effective oral hygiene with the preservation of restorative materials.

The observed differences in surface roughness among the toothpaste groups can be attributed, in part, to the physical and chemical characteristics of the abrasive agents, particularly the activated charcoal particles. The shape, size, and hardness of these particles play a critical role in determining their abrasivity. Irregularly shaped or sharp-edged particles are more likely to cause micro-abrasions on ceramic surfaces compared to those with smoother morphologies. Additionally, toothpaste excipients such as humectants, thickeners, and surfactants may influence how abrasive particles interact with the ceramic surface by modifying their dispersion, lubrication, and retention during brushing. Although charcoal is often promoted for its adsorptive properties, its potential chemical interactions with ceramic materials remain underexplored. Activated charcoal may adsorb surface ions or interact with the ceramic matrix in ways that alter surface energy, contributing to increased wear. From a clinical perspective, while the study was conducted in vitro, surface roughness above 0.2 µm has been associated with increased plaque accumulation, biofilm formation, and discoloration in vivo. Therefore, the increased roughness observed—particularly with Colgate Optic White with Charcoal—could translate into clinically significant consequences, including aesthetic degradation and compromised hygiene around ceramic restorations over time [31,32]. The results of the present study underscore the importance of careful toothpaste selection for patients with lithium disilicate restorations. Given that charcoal-containing toothpastes are often marketed for their whitening benefits, their abrasive potential may compromise the integrity of ceramic restorations, thereby increasing the likelihood of plaque retention, staining, and aesthetic deterioration over time. Based on the findings, patients with CAD/CAM lithium disilicate restorations should be advised to avoid highly abrasive formulations, particularly Colgate Optic White with Charcoal, to preserve surface smoothness and prolong restoration longevity.

Even though the present study specifically evaluated the effect of charcoal toothpaste on dental ceramic surfaces, its findings align with prior research that assessed the surface effects of charcoal toothpaste on human and bovine teeth. A recent in vitro study investigated the impact of various toothpastes, including carbamide peroxide and activated charcoal, on bovine teeth. The study compared conventional toothpaste, activated charcoal toothpaste, two carbamide peroxide toothpastes, and a combined carbamide peroxide and charcoal toothpaste. Surface roughness was measured before and after 420 brushing cycles. The results showed that charcoal toothpaste significantly increased surface roughness from 0.401 µm to 0.601 µm. Additionally, the combination of carbamide peroxide and charcoal further exacerbated surface roughness [32]. Another recent study on human teeth evaluated the effects of fluoride toothpaste, charcoal toothpaste, and whitening toothpaste on enamel surfaces. After 2500 brushing cycles, the findings indicated that both charcoal and whitening toothpastes caused greater increases in enamel surface roughness compared to fluoride toothpaste. The authors concluded that long-term use of charcoal and whitening toothpastes could negatively impact enamel smoothness, leading to a rougher surface texture over time [33]. These studies highlight the potential abrasive effects of charcoal-containing and whitening toothpastes, which may compromise the integrity of both natural and artificial dental surfaces with prolonged use. The findings underscore the importance of caution in selecting toothpaste formulations, particularly for patients seeking long-term oral health and preservation of dental restorations.

Despite the valuable insights provided by the present study, several limitations must be acknowledged. First, the study was conducted using flat lithium disilicate specimens, which may not fully replicate the complex geometry and occlusal wear patterns found in clinical restorations. In a real clinical setting, dental restorations are subject to dynamic forces and varied contact points, which may cause wear patterns that differ from those observed in flat, static specimens. As a result, the findings may not fully reflect the impact of charcoal-containing toothpastes on the long-term performance of restorations in a more realistic, clinical context. Additionally, the study focused on only four specific toothpaste formulations, which limits the scope of the findings. The market for whitening and charcoal-based toothpastes is vast, with many other products that vary in abrasivity and chemical composition. We also recognize that using distilled water as a diluent does not fully mimic the buffering and biochemical properties of saliva. Thus, the results may not be representative of the effects of all commercially available charcoal toothpastes. Future research should include a broader range of toothpaste formulations to account for these variations. Moreover, the study was conducted under controlled, short-term conditions, so it would be valuable to assess the long-term effects of charcoal toothpastes in simulated oral environments, where factors like salivary flow, temperature fluctuations, and dietary influences are also considered. Further studies should also explore additional mechanical properties, such as gloss retention, microhardness, and wear resistance, to provide a more comprehensive understanding of the effects of daily brushing on CAD/CAM lithium disilicate ceramics over time.

## 5. Conclusions

The present study demonstrated that charcoal-containing toothpaste formulations significantly influence the surface roughness of CAD/CAM lithium disilicate ceramics, with Colgate Optic White with Charcoal exhibiting the highest roughness values. These findings align with previous studies on the abrasive effects of whitening toothpastes and highlight the need for clinical recommendations favoring low-abrasiveness formulations for patients with ceramic restorations. Future studies should further investigate the long-term impact of toothpaste abrasiveness under dynamic oral conditions to enhance clinical guidelines for the maintenance of CAD/CAM lithium disilicate restorations.

## Figures and Tables

**Table 1:** Comparison of surface roughness among whitening-toothpaste groups.

Group & Roughness Measurement	Mean	Standard Deviation
Control: conventional toothpaste	540.70	21.68
Crest 3D White Charcoal	294.88	11.49
Colgate Optic White with Charcoal	1157.00	52.85
Arm & Hammer Charcoal White	593.37	37.69
*p*-value for Kruskal–Wallis test	<0.001	

**Table 2:** Post hoc analysis between whitening-toothpaste groups.

Sample 1–Sample 2	*p*-Value
Crest–Control	0.222
Crest–Arm & Hammer	0.002
Crest–Colgate	<0.001
Control–Arm & Hammer	0.701
Control–Colgate	0.002
Arm & Hammer–Colgate	0.222

## Data Availability

The original contributions presented in this study are included in the article. Further inquiries can be directed to the corresponding authors.
